# Cardiotoxicity of Non-Anthracycline Cancer Chemotherapy Agents

**DOI:** 10.3390/jcdd9030066

**Published:** 2022-02-23

**Authors:** Alexandros Briasoulis, Angeliki Chasouraki, Alexandros Sianis, Nikolaos Panagiotou, Christos Kourek, Argyrios Ntalianis, Ioannis Paraskevaidis

**Affiliations:** 1 Department of Clinical Therapeutics, National Kapodistrian University of Athens, 11528 Athens, Greece; angchasouraki@gmail.com (A.C.); alexsianis@gmail.com (A.S.); npanagiotou1997@gmail.com (N.P.); chris.kourek.92@gmail.com (C.K.); arg_nt@yahoo.gr (A.N.); iparask1@yahoo.gr (I.P.); 2 Division of Cardiovascular Diseases, University of Iowa Hospitals and Clinics, Iowa City, IA 52245, USA

**Keywords:** cardiotoxicity, cardiac dysfunction, non-anthracycline agents, chemotherapy, immunotherapy, cardioprotection

## Abstract

Throughout the last decades, newly developed chemotherapeutic agents and immunotherapies that target signaling pathways have provided patients with better prognoses, improved their quality of life and increased survival rates, thus converting cancer to a stable chronic disease. However, non-anthracycline cancer chemotherapy agents and immunotherapies including human epidermal growth factor receptor 2 (HER2) inhibitors, vascular endothelial growth factor (VEGF) inhibitors, Bcr-Abl tyrosine-kinase inhibitors (TKI), proteasome inhibitors, immune checkpoint inhibitors and chimeric antigen receptor T cells (CAR-T cells) may cause cardiovascular toxicity events and complications that usually interrupt the continuation of an appropriate treatment regimen, which induces life-threatening risks or leads to long-term morbidity. Heart failure, cardiac arrythmias and cardiomyopathies are the most common cardiovascular events related to cardiotoxicity due to chemotherapy. Each patient should be carefully assessed and monitored before, during and after the administration of chemotherapy, to address any predisposing risk factors and the new onset of cardiotoxicity manifestations early and treat them appropriately. The development of novel anticancer agents that cause minimal cardiovascular toxicity events or novel agents that ameliorate the adverse effects of the existing anticancer agents could drastically change the field of cardio-oncology. The aim of this narrative review is to demonstrate new knowledge regarding the screening and diagnosis of non-anthracycline-induced cardiotoxicity and to propose protective measures that could be performed in order to achieve the delivery of optimal care.

## 1. Introduction

Over the last few decades, due to the immense progress in the field of cancer treatment, there has been an important prolongation in life expectancy of patients diagnosed with malignancies. However, now that traditional cytotoxic chemotherapy along with molecular-targeted therapies and immunotherapy have improved the survival rates, it is very crucial that the off-target adverse effects are also given the proper attention [1]. In particular, complications of the cardiovascular (CV) system are considered significant, and they usually lead to higher morbidity and mortality rates of patients. 

Cardiotoxicity is defined as every CV event related to the use of cancer medication. The diagnostic criteria are the same as those used for the common population apart from the cardiac dysfunction associated with antitumor therapy, which is defined as a decrease in a left ventricular ejection fraction (LVEF) > 10% from baseline to a final LVEF below the lower limit of 53% [2]. Additionally, cardiotoxicity is divided into two different types in respect to reversibility, with reversibility referring to the recovery of cellular or organ function. Thus, type I cardiotoxicity is considered irreversible due to the cumulative administrated dose that causes myocardial cell loss, with anthracyclines being the most representative agents in this category [3]. In contrast, type II cardiotoxicity is not considered dose dependent and organ dysfunction can be reversed upon cessation of the treatment segment, with trastuzumab being the best representative agent [4]. 

It is very important for patients with cancer to be stratified according to their risk of developing CV toxicity, so that physicians can take precautionary measures and manage the delivery of the most appropriate cancer treatment. Although many stratification scores have been proposed for these patients, none of them are established yet. Patients considered as high risk for developing CV toxicity are shown in Table 1 [5]. Aside from the cytotoxic chemotherapy such as anthracyclines, alkylating agents and antimetabolites, the most representative target therapies that are also correlated with cardiotoxicity are human epidermal growth factor receptor 2 (HER2) inhibitors, vascular endothelial growth factor (VEGF) inhibitors, Bcr-Abl tyrosine-kinase inhibitors (TKI), proteasome inhibitors, and immune checkpoint inhibitors. 

**Figure 1:**
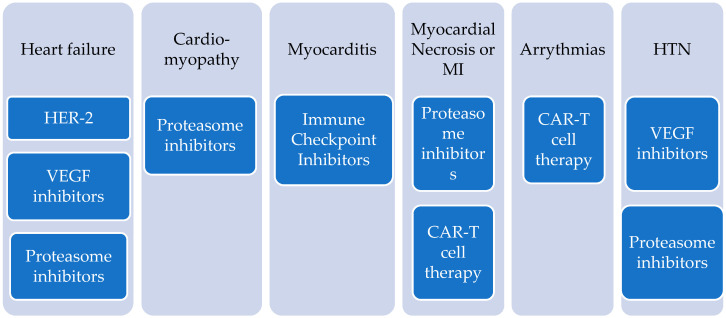
Most common manifestations of cardiotoxicity caused by non-anthracycline agents and the classes most often associated with them/that are implicated the most.

**Figure 2:**
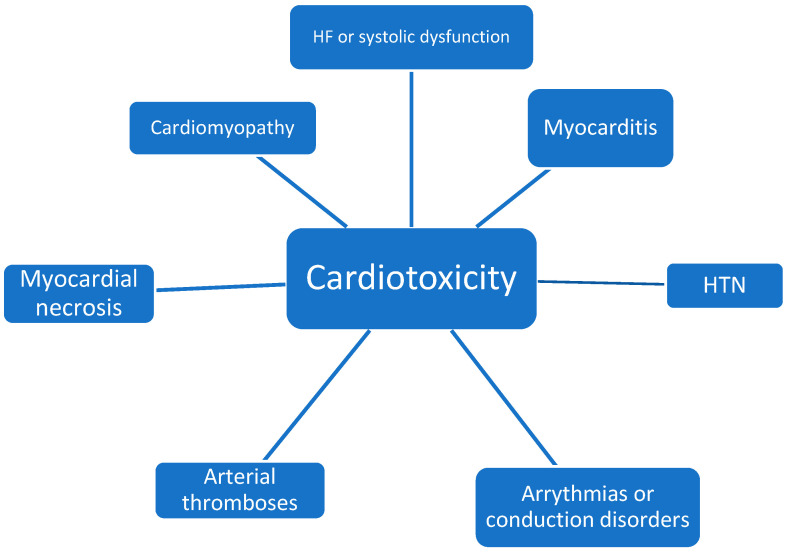
The most common cardiac complications of treatment with non-anthracycline agents.

The aim of this narrative review is to discuss the main diagnostic and prognostic aspects of non-anthracycline cancer chemotherapy agents that induce cardiotoxicity, focusing most on cardiomyopathies, and to highlight primary and secondary prevention strategies (Figure 1 and Figure 2).

## 2. HER-2 Targeted Therapies

About 15% of all breast cancers are human epidermal growth factor receptor 2 positive, which indicates a poorer prognosis [6,7]. Anti-HER 2 treatment is associated with cardiac dysfunction through impaired proliferating signals [7]. Even though the exact mechanisms are still unclear, experiments indicate that myocardial cell death is erbB2-dependent, since treatment that blocks this pathway can lead to mitochondrial dysfunction and the formation of reactive oxygen species [8].

Three percent to seven percent of patients treated only with trastuzumab, a recombinant human monoclonal antibody against HER2, may manifest some sort of cardiac toxicity [6]. A review of seven clinical trials reported cardiotoxicity events including congestive heart failure and reduced LVEF after trastuzumab administration [9]. Anthracycline monotherapy resulted in a lower incidence of cardiac dysfunction compared to combined therapy with trastuzumab [6,9]. The occurrence of cardiotoxicity events was irrelevant of whether trastuzumab was used as a first line treatment or for metastatic breast cancer [6]. Based on five trials of women with breast cancer, 5.9% of trastuzumab-treated women experienced a reduction in their LVEF compared to 2% of women in the control group [9]. Although the trastuzumab label suggests cardiac evaluation before, during and after administration, we should be prepared to manage drug-induced cardiac dysfunction that may occur [6]. As suggested in a previous study, early administration of b-blockers or angiotensin-converting enzyme (ACE) inhibitors may prevent cardiotoxicity in the setting of treatment with trastuzumab [10]. The American Heart Association proposes cardiovascular evaluation during treatment with HER-2 inhibitors, with the assessment of blood pressure, blood tests, echocardiography, and further evaluation, if needed based on these exams, every 3 months [5]. Cardiovascular abnormalities are thought to repair after discontinuation of anti-cancer treatment and heart failure (HF) therapy [11]. The United States Food and Drug Administration (FDA) suggests a 4 week discontinuation of trastuzumab if the LVEF drop is over 16% of the initial values or an absolute 10% fall occurs [12].

## 3. VEGF Inhibitors and Bcr-Abl TKIs

VEGF inhibitors play an important role in anticancer treatment as they inhibit angiogenesis, an essential function for the growth and metastasis of tumors [13]. Against the anticipation that endothelium’s dormancy would prevent any adverse effects, cardiovascular toxicity events have been documented during treatment with VEGF inhibitors [13]. According to a recent meta-analysis, severe hypertension was reported in 7.4%, coronary ischemia in 1.7%, arterial thromboembolism in 1.8% and cardiac dysfunction in 2.3% of patients receiving VEGF inhibitors [14]. The risk of developing HF during treatment with TKI inhibitors is reported to be between 1.5% and 4.1% along with bevacizumab with a 5-fold increase [15]. Congestive heart failure was reported in 8% of patients receiving sunitinib while 28% of chemotherapy-treated patients experienced a >10% decrease in LVEF [16]. In a meta-analysis of 10,647 patients, 2.39% of those treated with TKIs experienced asymptomatic left ventricular systolic dysfunction (LVSD) compared to 0.75% of those not treated with TKI [17]. Several trials have also recorded myocardial ischemia during treatment with VEGF inhibitors and TKIs [13].

Coronary artery disease, arterial hypertension, aortic stenosis, hypertrophic cardiomyopathy and obstructive sleep apnea are considered as risk factors for the development of cardiovascular adverse events during VEGF inhibitors treatment [14]. Findings that might be prognostic for cardiovascular toxicity are imaging abnormalities in echocardiograms and cardiac magnetic resonance (CMR), changes in electrocardiograms, and increased laboratory markers such as d-dimers and B-type natriuretic peptide (BNP) [10]. Angiogenesis is normally induced by hypoxia, ischemia and increased afterload such as augmented vascular resistance [14]. Vasoconstriction due to VEGF inhibitors is considered to be one of the main cardiotoxic mechanisms in hypertension. It can be attributed to decreased NO or increased endothelin-1 (ET-1) levels, causing less vasodilation and more vasoconstriction, respectively [13]. The inhibition of auto/intracrine VEGF signaling of endothelial cells may compromise their survival ability, contributing to cardiotoxicity mechanisms [14]. Studies have shown that these cardiovascular toxicity events are reversible after treatment cessation [13,14].

Hypertension is reported as an adverse event in most studies with VEGF inhibitors. It is dose-dependent and there is an increase in the risk when combination treatment is administered [13]. Cardiovascular assessment is proposed every 3 months for the first year and every 6 months during the rest of the treatment. Blood pressure monitoring is also very important, as well as training patients to measure their blood pressure at home [5]. Anti-hypertensive therapy with ACE inhibitors is usually sufficient. Discontinuation of therapy was necessary in 1.7% of patients receiving bevacizumab [18].

## 4. Proteasome Inhibitors

Proteasome inhibitors are mainly used to treat multiple myeloma. They include bortezomib, carfilzomib and ixazomib [10]. Carfilzomib irreversibly inhibits proteasome in about 80% of patients [19]. Reported cardiovascular toxicity events related to carfilzomib include arrythmia in 13.3% of patients, heart failure in 7.2%, ischemic heart disease in 3.4% and cardiomyopathy in 1.7%. Thirty-seven thromboembolic events and hypertension were also observed [19].

The mechanism of cardiovascular adverse events in patients receiving proteasome inhibitors is not yet fully understood. However, oxidative stress and endothelial dysfunction are thought to play an important role [20].

According to a meta-analysis of 24 studies, hypertension is the most common adverse event in the treatment with carfilzomib; it was reported in 12.2% of patients and was followed by congestive heart failure in 4.1% [20]. The ENDEAVOR study compared patients with relapsed or refractory multiple myeloma treated with carfilzomib and dexamethasone versus bortezomib and dexamethasone. Grade 1 and 2 hypertension was observed in 16% of patients in the first group compared to 6% of patients in the latter. Grade 1 and 2 cardiac failure was reported in 3% of the first group and Grade 3 was reported in 4%, whereas in the second group it was reported in 1% of each group. Ischemic heart disease was documented in <1% of both groups [21].

Before initiation of treatment with carfilzomib, acquiring a medical history and an assessment of established cardiovascular risk factors is suggested. During therapy, a comparison of BNP fluctuation with the baseline could warn us of the necessity of more frequent cardiological evaluations. To prevent cardiotoxicity, less IV fluid volume in each treatment session is proposed. If acute or chronic HF, hypertension or LVEF reduction occur, guidelines indicate cessation of treatment until recovery to baseline. These adverse events are thought to be reversible after chemotherapy cessation and proper treatment [22].

## 5. Immunotherapy 

### 5.1. Immune Checkpoint Inhibitors

Immune checkpoint inhibitors (ICIs) are monoclonal antibodies that re-enable the immune system to destroy cancer cells through inhibition of CTLA-4, PD-1 and PD-L1 pathways [23]. CTLA-4 antagonizes CD28 in binding to B7, causing inhibition of T cells. PD-1 binds to PD-L1, causing inhibition of cytotoxic T cells [24]. Cancerous cells exploit these pathways to evade the immune system and proliferate. Until now, seven agents have been approved in anticancer treatment; 1 CTLA-4 inhibitor—ipilimumab, 3 PD-1 inhibitors—pembrolizumab, nivolumab, and cemiplimab, and 3 PD-L1 inhibitors—atezolizumab, avelumab, and durvalumab [23]. These agents are indicated for 12 different hematological and solid cancers [25].

Activation of the immune system during immunotherapy may bring on immune-related adverse events (irAEs). The Common Terminology Criteria for Adverse Events classifies irAEs as low grade, high grade and lethal, and they are reported in 70% to 90% of patients [23]. Several organ systems may manifest irAEs with an incidence >10%, such as colitis, pneumonitis, dermatitis, hepatitis and endocrinopathies [26,27]. Cardiovascular toxicity events are less frequent with a reported incidence of 2.09%, including myocarditis, pericardial disease, arrhythmia, cardiomyopathy, conduction abnormalities, acute coronary syndrome and systemic vasculitis [23,24]. Escudier et al. reported that half of the patients with left ventricular systolic dysfunction after ICI therapy showed total reversibility, which was mostly attributed to corticosteroid therapy [28].

Although the exact mechanism of these cardiovascular irAEs is not yet completely understood, several hypotheses have been arisen. Studies in mice have confirmed the cardioprotective action of CTLA-4 and PD-1 pathways, and therefore, their inhibition may cause cardiac injury. Cytokine release from activated T cells is also thought to participate in this process. Furthermore, common high frequency T cell receptor sequences have been detected in both cardiac and tumor cells, suggesting the hypothesis of a cross-reaction mechanism [27,29].

Studies on the risk factors for developing cardiovascular adverse events are limited. Patients who received statins, angiotensin-converting enzyme inhibitors or aldosterone receptor blockers before immunotherapy are thought to have a higher risk of developing cardiovascular irAEs [25]. Hypertension and smoking history may also play an important role [25]. In a study of 30 patients with ICI-associated myocarditis, 23% of patients had concomitant myositis resulting in higher mortality rates in 51.7% of patients suffering from both compared to 14.9% of patients suffering from myositis alone [23,28]. One of the most strongly related risk factors in cardiac irAEs is combination therapy with anti-CTLA-4 and anti-PD1 agent. It has been shown that 28.9% of patients receiving this combination experienced myocarditis [30]. Mortality was estimated at 65.6% in patients receiving combination therapy compared to 44.4% in patients receiving monotherapy [27]. In a database study, the danger of developing myocarditis was about 4.5-fold higher in patients receiving combination therapy compared to monotherapy [31].

### 5.2. Myocarditis

The presentation of ICI-associated myocarditis may vary from mild symptoms such as fatigue and myalgia to more severe such as chest pain, dyspnea due to heart failure, pulmonary edema, arrhythmias, and sudden cardiac death [24,25,27,32,33]. In a multi-center study of 140 patients, 35 of which developed ICI-associated myocarditis, the median time of onset was 34 days (IQR 21–75 days) after the first ICI administration. Eighty-one percent of the cases experienced myocarditis within the first trimester of ICI treatment [30]. Thirty patients with ICI-associated cardiotoxicity from two cardio-oncology units presented symptoms in 65 days after the initiation of ICIs, with the time ranging from 2 to 454 days [28]. 

Due to the non-specific presentation of myocarditis, high clinical suspicion is necessary for its diagnosis. The first steps include laboratory work-ups with troponin and BNP/NT-proBNP. Ninety-four percent of patients that presented with myocarditis in a multicenter study had elevated troponin levels. High final/discharge troponin was strongly associated with MACE events (*p*-value = 0.002) [30]. Escudier et al. found that all patients had elevated BNP/NT-proBNP [28]. However normal values do not rule out the diagnosis of myocarditis [25].

Electrocardiograms and echocardiograms are also helpful, easily accessible, and non-invasive diagnostic tools. Common ECG findings are wide QRS/ST, T-wave inversion, abnormal Q, arrhythmias, and conduction disturbances [29]. Mahmood et al. recorded abnormal ECGs in 89% of patients [30]. The mean LVEF was 35% in 30 patients with ICI-associated myocarditis, ranging from 15 to 73%. Forty-six percent of these patients had an LVEF lower than 35% [28]. Patients with preserved and reduced LVEF had a 1.5-fold and 4.4-fold higher risk of MACE, respectively [27]. 

CMR is the preferred imaging test since it provides high quality imaging and useful tissue information [25]. According to the Lake Louise Criteria, myocardial edema and non-ischemic myocardial injury are required to diagnose myocardial inflammation. T2-mapping provides information for the first and abnormal T1, and late gadolinium enhancement (LGE) or extracellular volume (ECV) confirm the second criterion. Regional or global hypokinesis provides supportive evidence [34]. In a recent study, 48% of patients had LGE with a variable pattern, which was not MACE-associated [35]. However, CMR performed on day 4 showed a strong association with the detection of LGE (*p*-value < 0.001) [35]. When CMR is contraindicated or the patient is unable to perform CMR, PET/CT could provide information about myocardial inflammation and anatomy [27].

The histopathologic analysis of myocardial tissue from patients with suspected ICI-associated myocarditis demonstrated typical findings noted in non-ICI myocarditis with a diffuse T cell predominant lymphocytic infiltration in the myocardium with a predominance of CD3+, CD4+, CD8+ T lymphocytes and CD68+ macrophages, occasionally with eosinophils and also myocardial necrosis or fibrosis [28]. The pathologic findings and presentation of ICI-associated myocarditis often resemble cardiac allograft rejection, and these common characteristics support the treatment of ICI-associated myocarditis with immunosuppressive medications [23]. In cases of ICI myocarditis where there remains a persistent clinical suspicion despite non-invasive testing, endomyocardial biopsy is the gold standard for the diagnosis of myocarditis. The right heart biopsy, ideally five specimens, shows T- lymphocyte infiltration [23]. Eighty-nine percent of patients had T-lymphocyte infiltration in a two-unit study [28]. Zhang et al. reported lymphocytic infiltration in 98% of patients, among whom 38% had late gadolinium enhancement [35]. Immunohistochemistry reveals CD3+, CD4+ and CD68+ positive T cell markers [24]. It should be performed in patients with negative or ambiguous imaging results but high clinical suspicion, although focal distribution may reduce its sensitivity [32].

Management of ICI-associated myocarditis is based on clinical experience instead of prospective studies. First, permanent discontinuation of ICI treatment is necessary especially when Grade 3 or 4 (according to ASCO) symptoms have occurred [24]. Immunosuppressive agents should be started as soon as possible. Methylprednisolone with a dosage of 1 g/day IV for 3 days followed by prednisone of 1 mg/kg is the recommended treatment algorithm [29]. In another study, earlier initiation and a higher dose of corticosteroids were associated with less major adverse cardiovascular events. Earlier initiation of corticosteroids could be more beneficial since higher doses could not overcome the benefits of early treatment [36]. Mahmood et al. also showed that patients who experienced MACE have received a smaller initial dose of corticosteroids [30]. The tapering corticosteroids over 4–6 weeks may start when LVEF and conduction abnormalities are normalized and symptoms are resolved [29]. Since ICI-associated myocarditis and cardiac allograft rejection share histopathology findings, other immunosuppressive agents used for treatment are mycophenolate, anti-thymocyte globulin, IVIG, plasmapheresis and infliximab [27]. These could be considered in patients refractory to corticosteroid therapy [25]. Infliximab is not used when heart failure is present because it could degrade the clinical outcome [29]. Additional guideline-derived supportive therapy such as heart failure therapy and anti-arrhythmic agents may be used [24]. Re-challenge of ICI treatment is reported by Escudier et al. in four patients who had no recurrent episodes of irAEs [28]. There are no specific guidelines and such a decision should be individualized and discussed by a multi-disciplinary team [29].

## 6. CAR-T Cell Therapy

Chimeric antigen receptor T cells is a therapy used for hematologic malignancies and result in increased T cell function [27]. One adverse effect of this treatment is cytokine release syndrome (CRS) which is attributed to higher-than-normal release of inflammatory cytokines such as interleukin-6 (IL-6), interferon gamma (IFNγ), tumor necrosis factor-a (TNF-α) and interleukin-1 (IL-1) [27]. Most specifically, IL-6 is thought to play an important role in drug-mediated cardiotoxicity since studies suggest that it is involved in the infection and inflammation-mediated myocardial injury [25]. The fact that the median time to CRS is 6 days whereas the median time to major adverse cardiovascular events (MACE) is 11 days could support this relationship [37]. Furthermore, CRS grade 3 and 4 were independently associated with MACE along with baseline creatinine [37].

MACE include new onset of cardiac arrhythmias, coronary or cerebrovascular ischemia, symptomatic heart failure and cardiovascular death [37]. In a retrospective study of 145 patients treated with CAR-T cell therapy, 7.5% of patients experienced cardiac arrhythmia and 15% experienced heart failure, with a mean 49 ± 14% LVEF when MACE occurred compared to 61 ± 9% at baseline [37]. In a study of 187 patients, cardiac arrhythmias were reported in 7% of patients and CAD was reported in 11%, while 10.3% of them showed a reduction in LVEF from 58% to 37% after CAR-T cell therapy, indicating cardiomyopathy [38].

The risk factors that are associated with cardiovascular adverse effects during CAR-T cell therapy include coronary artery disease, CRS grade 3 and 4 and older age [38]. Patients with history of cardiovascular risk such as patients treated with statin, insulin and aspirin have a higher incidence of MACE [37]. Before the initiation of CAR-T cell therapy, studies propose a cardiovascular work-up including the patient medical history, ECG, and echocardiogram for all [25].

## 7. Cardioprotection

Strong interest has emerged to further examine the role of cardioprotective strategies and minimize treatment-related cardiotoxicity. In this context, various cardioprotective strategies have been evaluated to prevent the development of chemotherapy-related cardiotoxicity, with mixed findings [39,40,41]. A large, randomized trial compared lisinopril vs. carvedilol in patients with breast cancer receiving trastuzumab. Both treatments resulted in fewer cardiotoxicity events compared to placebo in patients receiving anthracyclines. Patients on placebo needed to interrupt their trastuzumab therapy more often than patients on preventive treatment with ACE inhibitors, ARBs or b-blockers [40]. The OVERCOME trial assigned patients with hematological malignancies to receive enalapril, carvedilol or placebo. Patients on enalapril or carvedilol presented less often with heart failure, LVEF < 45% or sudden cardiac death compared to the placebo group [41]. A previous meta-analysis of cancer patients with recent chemotherapy across 17 studies, showed significant benefits from the use of neurohormonal therapy with higher LVEF and better LV strain on follow up and no changes in other LV parameters in patients receiving chemotherapy. The absolute benefit in attenuating declines in LVEF was less than 5% and could be explained by inter-test variability [42]. These modest treatment effects on LVEF were consistently observed in trials examining strategies with renin-angiotensin-aldosterone-system inhibitors and beta-blockers and in the large subgroup of trials that exclusively examined breast cancer patients and those on anthracycline-based chemotherapy. Furthermore, there were numerically, but with statistical significance, fewer major clinical adverse events in the neurohormonal therapy arm. A more recent meta-analysis of nine randomized controlled trials [43] (n = 1362, all females) demonstrated that beta-blockers and ACEI/ARBs attenuated the decline in LVEF during trastuzumab and anthracycline treatments (with a mean difference of 2.4 and 1.5, respectively). Compared with placebo, LVEF was significantly higher in patients assigned to beta-blockers or ACEI/ARB on trastuzumab but not on anthracyclines. Recently, in a large double-blind, multicenter, placebo-controlled trial of 468 women, cardiotoxicity and treatment interruptions in patients with HER2-positive breast cancer treated with trastuzumab for 12 months were evaluated over a two-year period. Patients were stratified by anthracycline use and then randomized to receive lisinopril, carvedilol, or placebo. In those patients with HER2-positive breast cancer treated with trastuzumab, both lisinopril and carvedilol prevented cardiotoxicity specifically among patients receiving anthracyclines [42]. Finally, the recent SAFE trial was a four-arm, randomized, phase 3, double-blind, placebo-controlled, national multicentric study conducted at eight oncology departments in Italy. Bisoprolol, ramipril, or both drugs compared with placebo were administered for one year from the initiation of chemotherapy or until the end of trastuzumab therapy in case of ERBB2-positive patients. At 12 months, 3D-LVEF worsening was significantly lower in the bisoprolol and ramipril combination patients and a significantly lower percentage showed a 10% or greater worsening of GLS in the cardioprotection arms [44].

In contrast to anthracycline and HER-2 targeted agents, limited data are available on cardioprotection from potential cardiac adverse events related to other molecularly targeted agents. These agents are generally newer drugs with lower rates of toxicity, often reversible adverse events and less experience of toxicities compared with anthracyclines and HER-2 targeted agents. Furthermore, patients at the highest risk for developing such cardiac toxicity are often excluded from clinical trials that evaluate efficacy and safety of newer anti-neoplastic agents. Nevertheless, general principles apply for minimizing the development of cardiotoxicity across all classes of anticancer agents, including the molecularly-targeted agents. Patients treated with these agents should be stratified according to the risk for cardiac events based on their comorbidities. Primary prevention measures should be emphasized and the data available for HER-2 targeted therapies’ cardioprotection should be extrapolated to these agents. Evaluation and monitoring of LVEF or other biomarkers should be considered on a case-by-case basis and the toxicity profile, patient, and disease characteristics should be considered when making decisions about the monitoring of adverse events. Typically, the LVEF monitoring parameters recommended in the prescribing information for individual agents should be followed and patients with underlying cardiovascular disease or those developing early signs of cardiotoxicity based on echocardiographic parameters and biomarkers may need more frequent follow-up testing. 

## 8. Future Perspectives and Conclusions

Cardiotoxicity from non-anthracycline chemotherapeutics could lead to the termination of chemotherapy and/or immunotherapy treatment resulting in reduced 1-year or 5-year survival and poorer outcomes. The primary goal of cardio-oncology teams is to ensure the uninterrupted completion of the appropriate treatment regimen. Each patient should be carefully assessed before the beginning of chemotherapy, so as to detect risk factors that could predispose them to adverse events from chemotherapy (Table 2), and should be monitored during therapy in order to early diagnose and address any complication of the treatment. Moreover, careful monitoring should continue after the cessation of the treatment as many chemotherapy-induced CV events may present years later, affecting the patient’s quality of life and reducing survival. Both the predisposing risk factors and the new onset of cardiotoxicity manifestations (such as HF and cardiomyopathy) should be immediately addressed and treated aggressively according to the guidelines.

The development of novel anticancer agents that cause minimal CV toxicity events or novel agents that ameliorate the adverse effects of the existing anticancer agents could drastically change the field of cardio-oncology. Until then, further research is needed in order to establish more appropriate protocols in defining, screening, diagnosing and treating cardiotoxicity which would lead to better clinical outcomes.

## Figures and Tables

**Table 1:** Patients with higher risk for cardiotoxicity.

High-dose anthracycline (e.g., doxorubicin ≥ 250 mg/m^2^, epirubicin ≥ 600 mg/m^2^) High-dose radiotherapy (≥30 Gy) where the heart is in the treatment field Lower-dose anthracycline (e.g., doxorubicin < 250 mg/m^2^, epirubicin < 600 mg/m^2^) or HERis or VEGFis or proteasomeis or Bcr-Ablis and presence of any of the following factors: o Age ≥60 y o Lower-dose radiotherapy (<30 Gy) where the heart is in the treatment field o ≥2 risk factors including: smoking, hypertension, diabetes mellitus, dyslipidemia, chronic renal insufficiency, and obesity Previous heart disease Elevated cardiac biomarkers before initiation of anticancer therapy

**Table 2:** Recommended work up of patients on cancer chemotherapy agents.

1. Assess patiet history for risk factors of cardiotoxicity
Any previous exposure to cardiotoxic drugs or therapies Pre-existing comorbidities (DM, HTN, systolic or diastolic dysfunction, metabolic disorders)
2. Screen every patient before the beginning of the regimen
BP measurement ECG Echocardiogram Imaging stress test Blood tests and cardiac biomarkers
3. Optimize any risk factor
Treat any pre-existing comorbidities per guidelines (antidiabetics, antihypertensives, statins) Encourage a healthy lifestyle Consider the prophylactic use of ACEi and/or b-Blockers in very high-risk patients
4. Choose the best regimen and closely monitor for cardiotoxic manifestations
Close collaboration between cardiologists and oncologists for choosing the best regimen Prefer agents with minimal cardiotoxic effects, at maximal tolerated dose Monitor for early signs of cardiotoxicity (recommended every 3 months after the initiation of cancer therapy) Treat aggressively at first signs of cardiovascular adverse events As a last resort, consider discontinuation of cardiotoxic anti-cancer agents
5. Continue monitoring after the completion of the regimen
Cardiotoxic effects of cancer chemotherapeutic agents can manifest even years after the discontinuation of treatment

DM = diabetes mellitus, HTN = hypertension, BP = blood pressure, ECG = electrocardiogram.

## Data Availability

Not applicable.
